# Decoding the usefulness of non-coding RNAs as breast cancer markers

**DOI:** 10.1186/s12967-016-1025-3

**Published:** 2016-09-15

**Authors:** Maria Amorim, Sofia Salta, Rui Henrique, Carmen Jerónimo

**Affiliations:** 1 Cancer Biology and Epigenetics Group, IPO Porto Research Center (CI-IPOP), Portuguese Oncology Institute of Porto (IPOPorto), Research Center-LAB 3, F Bdg, 1st floor, Rua Dr. António Bernardino de Almeida, 4200-072 Porto, Portugal; 2 Institute of Biomedical Sciences Abel Salazar, University of Porto (ICBAS-UP), Porto, Portugal; 3 Department of Pathology, Portuguese Oncology Institute of Porto, Porto, Portugal; 4 Department of Pathology and Molecular Immunology, Institute of Biomedical Sciences Abel Salazar, University of Porto (ICBAS-UP), Porto, Portugal

**Keywords:** Biomarkers, microRNA, Long nonconding RNA, Diagnostic, Prognostic

## Abstract

Although important advances in the management of breast cancer (BC) have been recently accomplished, it still constitutes the leading cause of cancer death in women worldwide. BC is a heterogeneous and complex disease, making clinical prediction of outcome a very challenging task. In recent years, gene expression profiling emerged as a tool to assist in clinical decision, enabling the identification of genetic signatures that better predict prognosis and response to therapy. Nevertheless, translation to routine practice has been limited by economical and technical reasons and, thus, novel biomarkers, especially those requiring non-invasive or minimally invasive collection procedures, while retaining high sensitivity and specificity might represent a significant development in this field. An increasing amount of evidence demonstrates that non-coding RNAs (ncRNAs), particularly microRNAs (miRNAs) and long noncoding RNAs (lncRNAs), are aberrantly expressed in several cancers, including BC. miRNAs are of particular interest as new, easily accessible, cost-effective and non-invasive tools for precise management of BC patients because they circulate in bodily fluids (e.g., serum and plasma) in a very stable manner, enabling BC assessment and monitoring through liquid biopsies. This review focus on how ncRNAs have the potential to answer present clinical needs in the personalized management of patients with BC and comprehensively describes the state of the art on the role of ncRNAs in the diagnosis, prognosis and prediction of response to therapy in BC.

## Background

Breast cancer (BC) is one of the most common cancers with more than 1,300,000 cases diagnosed and 450,000 deaths occurring each year, worldwide [1]. Due to earlier diagnosis and implementation of adjuvant chemo- and hormone-therapies (HT), BC mortality has been declining, although it remains the most common cause of cancer-related death among women [2]. At present, most patients are diagnosed at localized disease stage, but 20–85 % of all patients will eventually develop recurrent and/or metastatic disease [3].

BC is intrinsically heterogeneous, representing a spectrum of diseases with distinct morphology, molecular traits, prognosis, and therapeutic options. On the basis of gene expression, BC cases are often classified into one of five intrinsic subtypes [4]. The large majority of estrogen receptor (ER) and/or progesterone receptor (PR)-positive (^+^) tumors are of the luminal subtypes that typically express luminal cytokeratins (CK) 8 and 18 [5]. These tumors are further subdivided into Luminal A and Luminal B, according to the expression levels of Ki67, a nuclear protein that is associated with cellular proliferation. The ER and PR-negative (^−^) tumors are divided into three subtypes: the basal-like subtype, characterized by the expression of CK 5/6 and CK17; the human epidermal growth factor receptor 2 (HER2)-enriched subtype, which are positive for HER2; and the “normal-like” subtype, characterized by a similar gene expression pattern as the normal breast. This last subtype remains enigmatic as to whether it represents a separate subtype or a technical artifact introduced by the contamination of cancerous cells with their surrounding normal tissue [5].

BC clinical decisions are based on routine assays for ER, PR and HER2, as well as Ki67 [6]. The molecular phenotype of the tumor is indicative of the most suitable treatment, i.e., either endocrine therapy (ET) in hormone receptor positive or HER-targeted therapy in HER2^+^ tumors [7]. Globally, ER^−^ tumors have a poorer prognosis in the first few years after diagnosis, but after 5–10 years, ER^+^ tumors demonstrate the poorest outcome [8]. However, not all ER^+^ BCs behave similarly, and the studies conducted in recent years show that luminal A and B BCs should be perceived as distinct entities [9]. Luminal A subtype has been shown to exhibit good clinical outcomes with ET whereas the pattern of mortality rates associated with the luminal B tumors is similar to those of the non-luminal subtypes [10]. However, Luminal A, the most frequently occurring BC subtype, is also the most heterogeneous subtype, both molecularly and clinically [11]. Indeed, ER expression itself fails to predict which ER^+^ tumors will respond or be resistant to different modalities of ET, and resistance has been reported in 30 % of ER^+^ BCs [12].

Due to molecular heterogeneity, clinical decisions based solely upon histopathologic analysis or one or small numbers of genes or their coding proteins in the tumor tissue are limited. Moreover, the widespread use of gene-expression profiling using commercially available molecular signatures for the examination of multiple expressed genes is also of limited application, primarily due to the cost and to reproducibility issues [13, 14].

Recently, several studies have reported on the association between microRNAs (miRNAs) and BC, suggesting its usefulness as disease biomarkers. Interestingly, miRNA detection in bodily fluids appear to have superior accuracy than messenger RNA (mRNA) profiling because of their high tissue-specificity, stability, and aberrant expression in different tumor types [15]. miRNAs have the additional advantage of being long-lived in vivo [16] and very stable in vitro [17, 18], which might be critical in a clinical setting. Indeed, tumor cells were suggested to release miRNAs stabilized by their association with RNA-binding proteins and by their incorporation into microvesicles [19, 20]. The emergence of non-coding RNA (ncRNAs) as biomarkers may add robustness to the current molecular classification of human BC, with the potential for improving diagnosis and monitoring of BC. Thus, in this review, we will focus on ncRNAs as potential diagnostic, predictive and prognostic biomarkers for BC management.

### Evidence acquisition

For the selection of bibliography, PubMed publications on BC were searched using the keywords breast cancer, noncoding RNAs and microRNAs. References of all articles were also examined for additional potentially relevant studies. The criteria for article selection were: written in English, central theme based on ncRNAs and BC. Original reports were selected based on the detail of analysis, mechanistic support of data, novelty, and potential clinical usefulness of the findings.

### Non-coding RNAS

It is currently acknowledged that at least 98 % of the mammalian genomes and other complex organisms are transcribed into ncRNAs [21]. In fact, ncRNAs that were previously thought to be “transcription noise”, are believed to be a hidden layer of internal signals that control various levels of gene expression, playing a significant role in cell homeostasis and its deregulation is involved in the development of several human diseases. The family of ncRNAs, in addition to the well knows transfer RNAs (tRNAs), ribosomal RNAs (rRNAs), and small nucleolar RNAs (snoRNAs), includes the recently discovered long noncoding RNAs (lncRNAs) and miRNAs.

### Transfer RNA (tRNA)

Transfer RNAs are small ncRNA transcripts, typically with 76–90 nucleotides (nt) in length, that serve as physical link between mRNA and the aminoacid sequence of proteins [22]. In 2009, Pavon-Eternod [23] analyzed genome-wide tRNA expression and found that tRNAs were increased in BC compared to normal breast tissues. Their results also suggested a functional consequence of tRNA over-expression in tumor cells, which seems to be selective and may increase the translational efficiency of genes relevant to cancer development and progression.

Recent studies indicated that precise cleavage of tRNAs generate active products [24]. Indeed, high levels of tRNA-derived miRNas or of tRNA-derived molecules termed 5′tRNA halves are likely to be a manifestation of tRNA over-expression. Park [25] reported that miR-1280—a tRNA-derived fragment was significantly up-regulated in blood of BC patients, particularly in metastatic BC patients, compared to healthy subjects and decreased significantly after systemic treatment in patients who responded to treatment, while increasing in the blood of patients with non-responding tumors. Moreover, BC is associated with expression deregulation—either increase or decrease—in the circulating levels of 5′tRNA halves derived from specific tRNA isoacceptors [26], and changes in circulating 5′tRNA halves were associated with specific tumor features, such as ER/PR/HER2-status, raising the possibility of a causal connection with some aspects of breast carcinogenesis.

### Long noncoding RNAs (lncRNAs)

LncRNAs are ncRNA molecules usually longer than 200 nts that do not fit into known classes of small or structural RNAs, and that may function as either primary or spliced transcripts [27]. LncRNAs may be transcribed from various genomic locations, as well as in their own stand-alone position in the genome—long intergenic non-coding RNAs (lincRNAs) [28]. LncRNAs have gained widespread attention in recent years as a potentially new and crucial layer of biological regulation, controlling cell cycle, apoptosis and differentiation by acting as protein-DNA or protein–protein scaffolds, miRNA sponges, protein decoys, and regulators of translation [29].

### LncRNAs in breast cancer

LncRNAs were already found to be differentially expressed in BC tissues compared to normal breast tissues and recent studies have demonstrated their key regulatory role in gene expression and BC biology through diverse mechanisms [30].

### Diagnostic biomarkers

Expression levels of lncRNAs have been investigated in BC tissues compared to normal tissues indicating that some may be potential biomarkers for BC diagnosis. Ding et al. found that lincRNA-BC2 and lincRNA-BC5 were consistently up-regulated (more than twofold) in BC samples, whereas lincRNA-BC4 and lincRNA-BC8 were down-regulated [31]. Moreover, lincRNA-BC4 expression was significantly lower in grade III BC, and lincRNA-BC5 expression was significantly higher in grade III, whereas lincRNA-BC2′ expression significantly associated with lymph node metastasis (LNM). Remarkably, lncRNAs’ expression was also found to be highly associated with BC subtype classification [32]. Later studies have also demonstrated that lncRNAs are amenable for detection in bodily fluids. For example, the serum expression levels of circulating lncRNA RP11-445H22.4 were found significantly increased in BC patients, identifying BC cases with 92 % sensitivity and 74 % specificity [33].

### Prognostic biomarkers

In addition to lncRNAs potential use as diagnostic biomarkers, they have been suggested as valuable prognostic biomarkers. Zhao and co-workers identified a set of lncRNAs that distinguished low-risk from high-risk BC patients [34]. Patients with significantly higher LINC00324 expression and lower PTPRG antisense RNA 1 (PTPRG-AS1) and small nucleolar RNA host gene 17 (SNHG17) expression showed longer overall survival (OS). In another study, high SPRY4 intronic transcript 1 (SPRY4-IT1) expression levels were also associated with poorer prognosis, specifically shorter disease-free survival (DFS) [35].

HOX transcript antisense RNA (HOTAIR) overexpression in BC tissues has been associated with higher invasion and metastatic capacities, and suggested as an OS and progression free-survival (PFS) biomarker [36]. Specifically, in ER^+^ BC patients, HOTAIR expression was shown to independently predict the risk of metastasis [37]. Similarly, metastasis-associated lung adenocarcinoma transcript 1’s (MALAT1) upregulation was found in primary BC and its levels were further increased in the respective metastases [38]. Conversely, BC040587 [39], neuroblastoma associated transcript 1 (NBAT1) [40] and eosinophil granule ontogeny transcript (EGOT) [41] were found downregulated in BC samples and associated with poor prognosis. Furthermore, LINC00472 high expression levels in BC tissues associated with less aggressive behavior and more favorable outcome [42].

### Predictive biomarkers

LncRNAs have been suggested as valuable predictive biomarkers. Indeed, BC anti-estrogen resistance 4 (BCAR4) overexpression has been shown to predict tamoxifen resistance [43]. On the other hand, lincRNAs LINC00160 and LINC01016 were both found highly overexpressed in ER^+^ tumors compared to ER^−^ tumors and normal tissues, being significantly associated with longer OS of luminal A BC [44]. Interestingly, these lincRNAs may identify patients that respond to ET, functioning as robust predictive biomarkers for ER activity.

Besides ET resistance, progression or recurrence due to resistance to trastuzumab or other commonly used therapeutic approaches, such as chemotherapy and radiotherapy, also constitute a significant clinical challenge. LncRNA activated by TGF-β (ATB) has been associated with trastuzumab resistance in BC patients [45]. Conversely, lncRNA colon cancer associated transcript 2 (CCAT2) overexpression identified a subset of BC patients that might not benefit from cyclophosphamide, methotrexate and fluorouracil (CMF) based adjuvant chemotherapy [46]. Finally, Chen et al. [47] demonstrated that overexpression of lincRNA Regulator of Reprogramming (ROR) is associated chemotherapy tolerance.

### microRNAs (miRNAs)

miRNAs are endogenous, small non-coding single-stranded RNAs with an approximate length of 22 nt, encoded by various genomic regions in either sense or antisense orientation [48]. miRNAs are critical for a wide range of biological processes exerting a finely tuned regulation of gene expression at posttranscriptional level [49].

### miRNAs in breast cancer

**Fig. 1:**
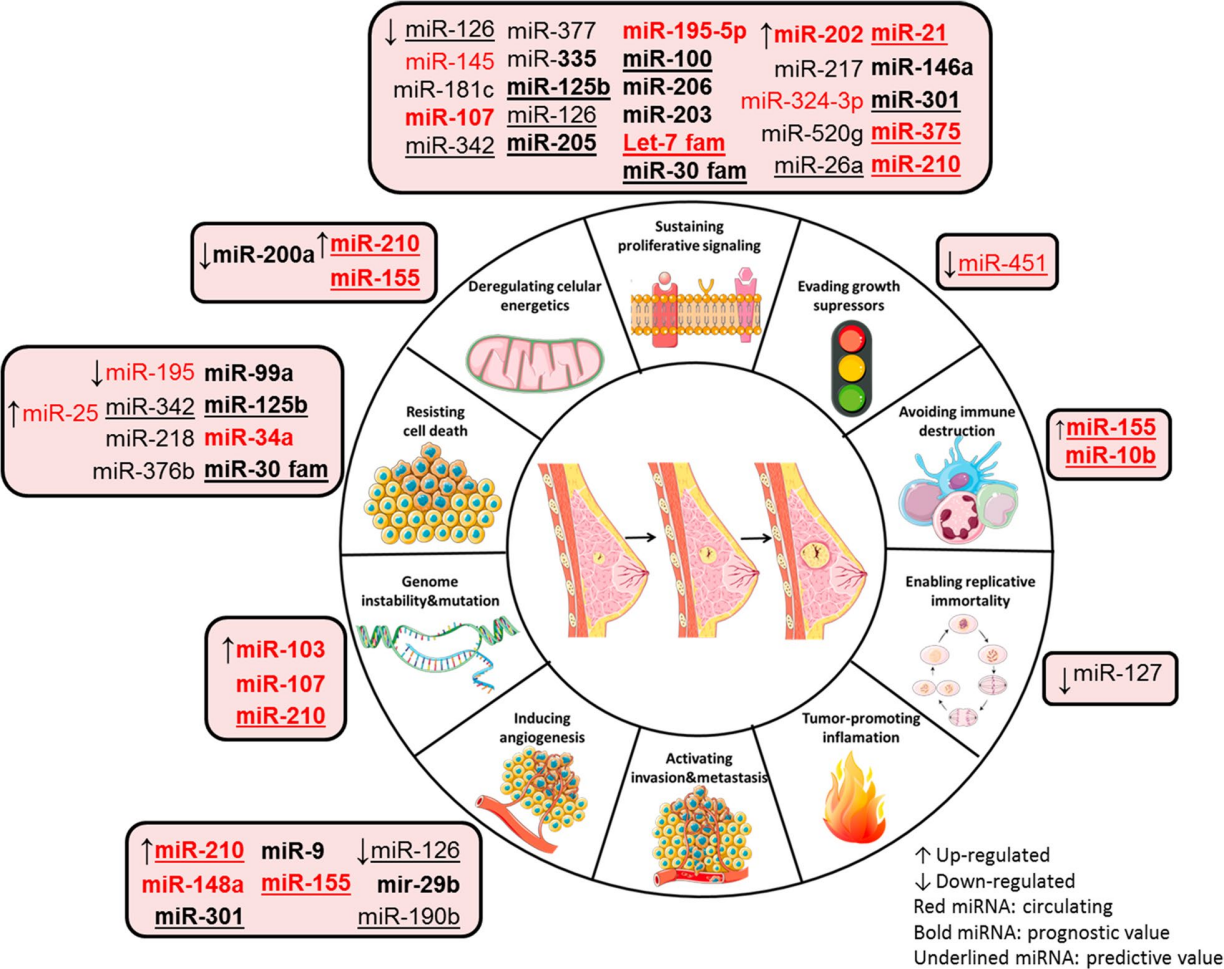
miRNAs as key regulators of BC hallmarks. Expression of miRNAs (↑up-regulated and ↓ down-regulated) grouped according to their function in the hallmarks of breast cancer: circulating miRNAs (*red*) and non-circulating miRNAs with prognostic (*bold*) and predictive (*underlined*) value. *miR* microRNA; *fam* family

miRNA dysregulation in cancer was first reported in 2002 [50]. Since then, the emergence of miRNAs has been one of the defining developments in cancer biology with several studies demonstrating a differential miRNA expression profile and global miRNA downregulation in human malignancies compared with paired normal tissues. Indeed, aberrant miRNA expression in human tumors is not just a casual association, as it exerts a causal role at different steps of the tumorigenic process. Some of the miRNAs that will be mentioned here have already been associated with several hallmarks of cancer [3, 51, 52] (Fig. 1).

miRNAs might be classified into oncogenic miRNAs (oncomiRs) or tumor suppressor miRNAs, depending on their targets. OncomiRs act by repressing the expression of tumor suppressor genes and are frequently upregulated in cancer. Tumor suppressor miRNAs act by targeting oncogenes and are frequently downregulated in cancer. However, this miRNA categorization may be inaccurate, as many studies have shown that miRNAs may present a dual function, with oncogenic or tumor suppressive properties based on tumor type and cellular context [53]. Furthermore, some miRNAs are consistently up- or down-regulated in tumor specimens, whereas other, such as miR-221 and miR-10b, exhibit a more irregular pattern of expression [54]. MiR-10b was found downregulated in all BCs from metastasis-free patients, but elevated miR-10b levels were found in primary tumors from patients harboring metastasis, suggesting that miR-10b might be differentially deregulated along tumor progression [55]. Volinia et al. [56] studied this change in miRNA expression along cancer progression and found that let-7d, miR-210 and miR-221 were downregulated in the ductal carcinoma in situ compared to normal breast tissue, but it was found to be upregulated in the transition to invasive carcinoma, featuring an expression reversal along the cancer progression path.

### Diagnostic biomarkers

Each tumor type has a distinct miRNA signature that distinguishes it from normal tissues and other cancer types [15]. In 2005, Iorio et al. [57] identified a 13-miRNA signature that could discriminate BC from normal breast tissues with perfect accuracy. Among the differentially expressed miRNAs, the most consistently dysregulated were miR-125b and miR-145 (downregulated), and miR-21 (up-regulated). Since then, many studies have looked at specific miRNAs dysregulated in BC with a diagnostic purpose.

In addition to studies of miRNA expression patterns in tissues, expression profiling studies of miRNAs in bodily fluids have been performed, to investigate whether bodily fluids could be used to differentiate BC patients from healthy individuals. In this context, Heneghan et al. [58] found significantly higher levels of miR-195 and let-7a in the blood of BC patients compared to healthy controls, detecting BC with high sensitivity and specificity. Several studies have also highlighted differences in the profiles of serum and plasma miRNAs in cancer compared to healthy individuals. MiR-222, for example, was significantly increased in the serum of BC patients [59], while higher miR-205 levels have been found in the sera of healthy individuals compared to BC patients [60]. Furthermore, Zhao et al. found that miR-195 was downregulated in the plasma of BC patients compared to healthy subjects [61].

miRNA profiles show better diagnostic performance as well as increased sensitivity than individual miRNAs, for BC detection. Hu et al. identified a 4-miRNA signature with increased concentrations in the serum of BC patients that could distinguish BC patients from healthy individuals with 92.1 and 93.4 % sensitivity and specificity, respectively [62]. More recently, Zhang and co-workers have found a 3-miRNA signature in serum as a diagnostic biomarker for non-invasive early detection of BC [63], whereas Ng et al. reported that the combination of miR-145 and miR-451 levels in plasma may discriminate normal individuals from BC patients, both at early and advanced stages of disease [64]. Finally, Cuk et al. have also found a panel of deregulated plasma miRNAs that were elevated in women with benign and stage I or II BC, that might be attractive candidates for early BC detection [65].

Table 1 summarizes these and others non-circulating and circulating miRNAs already described and validated in large cohorts for BC diagnosis.

**Table 1:** Non-circulating and circulating miRNAs for BC diagnosis

	miRNAs	Sample	Validation techniques	Samples size	Sensitivity	Specificity	AUC	Refs
Non-circulating miRNAs	↑miR-23a	BC tissues	qRT-PCR	76BC *vs*. 36 benign *vs*. 36 N	0.829	0.100	0.915	[66]
	↑miR-155, -21, -184, -518b, -572, -601, -622 ↓miR-125b		TaqMan qRT-PCR	24BC vs. 6 N	–	–	–	[67]
	↑miR-660-5p, ↓miR-99b-5p, -574-3p, -769-5p		SYBR Green qRT-PCR	56BC vs. 9 N 60BC vs. 11 N	–	–	–	[68]
Circulating miRNAs	↑miR-222	Serum	qRT-PCR	50BC vs. 50 N	0.74	0.60	0.671	[59]
	↑miR-16, -25, -222, -324-3p		TaqMan qRT-PCR	76BC vs. 76 N	0.921	0.934	0.928	[62]
	↑miR-145, -155, -382		qRT-PCR	61BC vs. 10 N	0.976	0.100	0.988	[69]
	↓miR-205		qRT-PCR	58BC vs. 93 N	0.862	0.828	0.84	[60]
	↑miR-199ª, -29c, -424		SdM-RT-PCR	76BC vs. 52 N	0.776	0.846	0.901	[63]
	↑miR-92a, miR-133a		qRT-PCR	132BC vs. 101 N	–	–	0.91	[70]
	↓miR-200c	Whole blood	qRT-PCR	57BC vs. 20 N	0.90	0.702	0.79	[71]
	↓miR-145 ↑miR-451	Plasma	TaqMan qRT-PCR	70BC vs. 50 N	0.900	0.920	0.931	[64]
	↑miR-127-3p, -148b, -376a, -376c, -409-3p, -652, -801		TaqMan qRT-PCR	120BC vs. 60 N	0.800	0.720	0.81	[65]
	↓miR-195		SYBR Green qRT-PCR	210BC vs. 102 N	0.69	0.892	0.859	[61]
	↑miR-16, -148a, -19b, -22a ↓Let-7d, let-7i, miR-103, -107		qRT-PCR	108BC vs. 88 N	0.91	0.49	0.81	[72]
	↑miR-505–5p ↑miR- 96–5p		qRT-PCR	114BC vs. 116 N	–	–	0.72 0.72	[73]

^*↑*^Up-regulated ^↓^ Down-regulated

*N* normal, *SdM* serum-direct multiplex

Despite the identification of non-circulating and circulating miRNAs aberrantly expressed in BC, discrepancies remain among the different miRNA signatures reported, probably due to differences in clinicopathological variables and the intrinsic heterogeneity of BC. Therefore, an attempt has been made to develop miRNA signatures that might reflect distinct histopathological features of BC.

Indeed, altered miRNAs levels that predict ER, PR and HER2 receptor status have already been identified (Table 2). Lowery et al. identified a 15-miRNA predictive signature corresponding to the expression of ER, PR, and HER2 receptor status [74]. Recently, Cizeron-Clairac and co-workers found that 20 miRNAs were significantly deregulated in ER^+^ compared to ER^−^ BCs [75]. Up-regulation of miR-1244 and downregulation of miR-30e were specific of ER^−^ tumors, whereas downregulation of miR-18a, miR-18b and miR-654-3p and up-regulation of miR-342-5p and miR-190b were specific of ER^+^ tumors.

**Table 2:** miRNAs which increased expression predicts for ER, PR and HER2 receptor status in BC

		Refs.
*ER status*		
ER^+^	miR-342, -217, -190b, -218, -342-5p	[74–76]
ER^−^	miR-299-3p, -190, -135b, -*182*, -*21*, -30e, -1244, -*10b*, -*375*	[58, 74, 75, 77, 78]
*PR status*		
PR^+^	miR-520f-520c, -377, -*155*	[74, 79]
PR^−^	miR-520 g, -527-518a, -*182*, -*10b*, -*375*, -*21*	[74, 77, 78]
*HER2 status*		
HER2^+^	miR-520d, -376b, -146a-5p, -*375*	[74, 80]
HER2^−^	miR-181c, -*122*	[74, 78]

Circulating miRNAS are represented in italic

Circulating miRNAs were also found to correlate with ER, PR and HER2 status in several studies. For example, higher levels of circulating miR-182 [77], miR-21 and miR-10b [58] have been correlated with ER/PR^−^ tumors. Furthermore, miR-155 expression levels were higher in sera of women with hormone-sensitive BCs [79]. Moreover, higher levels of circulating miR-375 were associated with ER/PR^−^ and HER2^+^ tumors, whereas higher levels of circulating miR-122 associated with HER2^−^ tumors [78].

Several specific miRNA expression profiles have also been associated with BC molecular subtypes. Iorio et al. identified a distinct miRNA signature in luminal BC, with overexpression of miR-191 and miR-26 and downregulation of miR-206 [57]. Likewise, miRNAs might differentiate between basal and luminal tumor subtypes in an independent data set [81]. In an attempt to capture the heterogeneity of Luminal A and Luminal B BCs, Endo et al. compared the expression profiles of miRNAs in ER^+^ tissues between ER^high^/Ki67^low^ tumors and ER^low^/Ki67^high^ tumors [82]. They found that six miRNAs (let-7a, miR-15a, miR-26a, miR-34a, miR-193b and miR-342-3p) were upregulated and a single miRNA was downregulated (miR-1290) in ER^high^/Ki67^low^ tumors [82].

### Prognostic biomarkers

miRNAs have been correlated with clinical and pathological features that associate with prognosis in different tumor types and subgroups of BC patients [83, 84]. The search for prognostic biomarkers is a continuous and fundamental work in progress, since patients at higher risk may require differential therapeutic interventions.

**Fig. 2:**
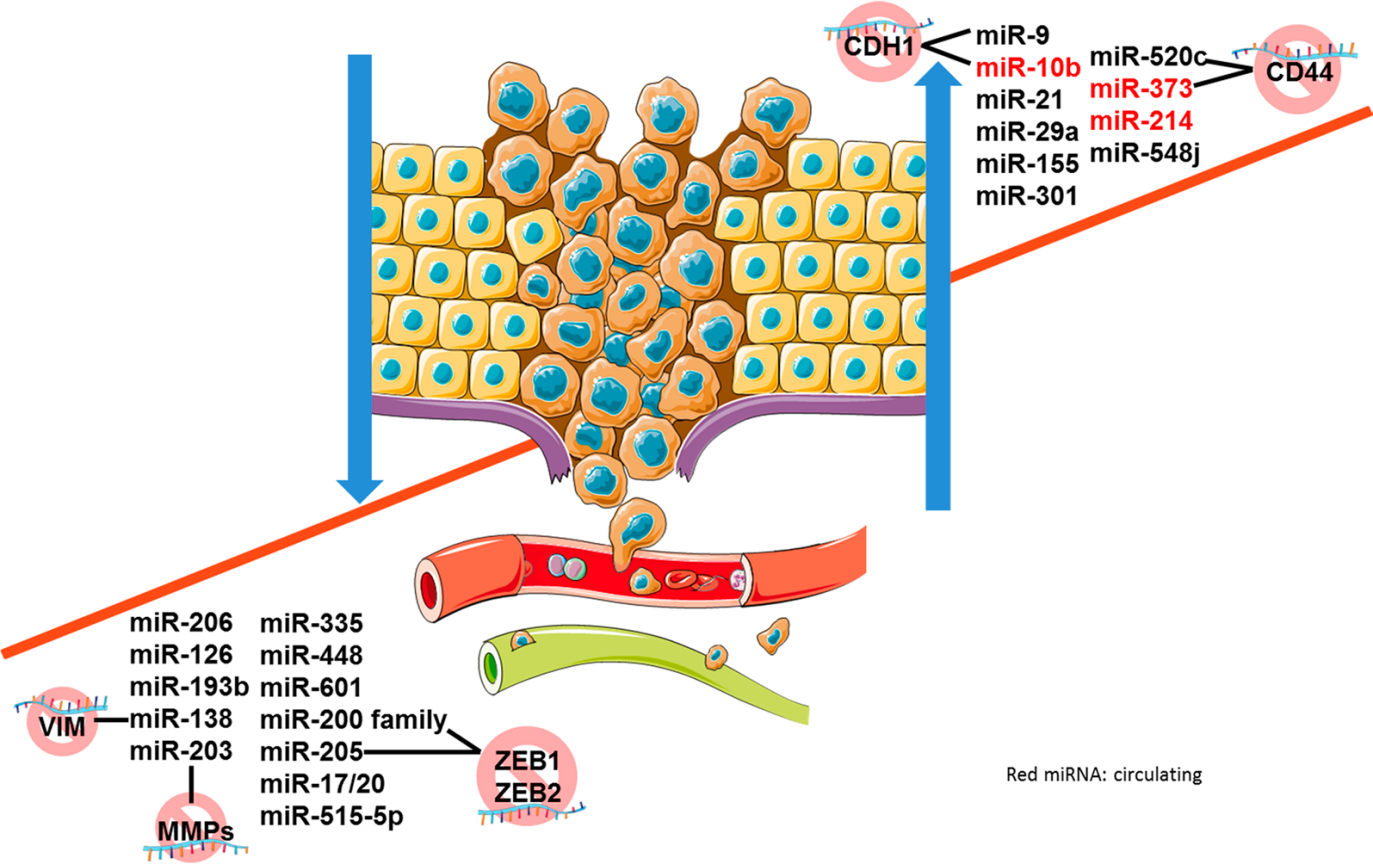
miRNAs and breast cancer metastasis. miRNAs are crucial in metastatic spreading, acting either as oncogenes, typically up-regulated, or as tumor suppressor genes, typically down-regulated. Circulating (*red*), non-circulating miRNAs and examples of targets. *miR* microRNA, *CD* cluster of differentiation, *ZEB* Zinc Finger E-Box binding homeobox 1, *MMP*s matrix metalloproteinases, *VIM* vimentin, *CDH1* cadherin 1

One of the main reasons for the BC associated mortality is metastization [85], a complex process that allows the primary tumor cells to spread to the neighboring as well as to distant parts of the organism. miRNAs appear to be involved in the phenotypic changes associated with metastasis formation, such as epithelial-mesenchymal transition, as well as with the presence of circulating tumor cells, which correlate with metastatic spread [86]. miRNAs may act either as promotors of BC metastasis or as metastasis suppressors. Metastasis promoters include miR-9 [87], miR-10b [55, 88], miR-21 [89], miR-29a [90], miR-155 [91], miR-520c [92], miR-373 [88, 92], miR-214 [93], miR-301 [94] and miR-548j [95], whereas metastasis suppressors include miR-17/20 [96], miR-126 [97], miR-193b [98], miR-206 [99], miR-335 [100], miR-448 [101], miR-601 [102], miR-138 [103], miR-515-5p [104], miR-203 [105], miR-200 family and miR-205 [106]. These specific miRNAs might serve as valuable biomarkers for predicting metastasis and tumor recurrence, which determine the unfavorable prognosis of BC patients. All these miRNAs were validated in tumor tissues and/or bodily fluids from BC patients and are depicted in Fig. 2.

miRNAs have also been associated with other clinical and pathological features that influence BC patients’ prognosis. miR-21, aside from being a driver of metastasis, has been known to create a pro-tumorigenic environment by targeting numerous tumor suppressor genes, and its overexpression was correlated with advanced tumor stage and poor OS and DFS in BC patients [107, 108]. Several studies have independently associated miR-210 with BC development and its expression levels were correlated with tumor aggressiveness and poor prognosis [109, 110]. Moreover, some miRNAs have been associated with a good prognosis, such as the miR-30 family, that has been identified as an individual favorable prognostic marker in several studies [111–113]. Other miRNAs, particularly downregulation of the miR-200 family, have also been associated with BC stem cells [114], one of the main obstacles for effective treatment of BC [115].

Some studies have focused on particular subtypes of BC. Bailey et al. evaluated miRNAs expression in ER^+^ BC tissues and found that a cluster comprising let-7c and miR-125b was uniformly low in luminal B and lost in a subset of luminal A patients with worse OS, indicating its potential as biomarker of good outcome in ER^+^ luminal A BC patients [116]. Gasparini and co-workers identified a 4-microRNA signature in triple negative BC that allowed for the stratification of those patients into high- and low-risk groups [117]. Up-regulation of miR-493 and miR-155 correlated with better patient outcome, whereas miR-30e and miR-27a downregulation correlated with worse outcome [117].

Interestingly, some miRNAs may differentially influence outcome depending on the characteristics of the tumors. Tuomarila et al. reported that high miR-200c expression independently predicted poor OS in patients with PR^−^ tumors, whereas low expression independently predicted poor OS in patients with PR^+^ tumors [118].

These and other miRNA signatures or single miRNAs that have been associated with prognosis are summarized in Table 3.

**Table 3:** miRNA panels or single miRNAs proposed with a prognostic aim

	miRNA	Biological sample	Consequences	Refs.
miRNAs associated with positive outcome	miR-100	Tumor tissues	↑OS	[119]
	miR-29c			[120]
	miR-181d, -195-5p			[80]
	miR-128			[121]
	Let-7b, miR-205		↑RFS, OS	[122]
	miR-342-5p			[123]
	miR-497		↓TNM, LNM	[124]
	miR-133a		↑RFS	[125]
	miR-30 family		↑ OS, RFS, DFS ↓Metastasis	[111–113]
	miR-206		↑OS ↓TNM, LNM	[126]
	miR-601		↓Metastasis ↑MFS	[102]
	miR-124		↑OS ↓TNM, LNM	[127]
	miR-138		↓TNM, LNM	[103]
	miR-190b		↑MFS, OS	[75]
	miR-200b		↓LNM	[128]
	miR-29b		↑DFS, OS	[129]
	miR-27a			[130]
	miR-374b-5p, -218-5p, -126-3p	TNBC tissues	↑DFS, OS	[131]
	miR-155 -493		↑OS	[117]
	let-7c, miR-99a, -125b	Luminal A BC tissues	↑OS	[116]
miRNAs associated with negative outcome	miR-21	Tumor tissues	↑Grade, TNM, LNM, metastasis ↓DFS, RFS, OS	[107, 108]
	miR-210		↓OS, RFS, DFS, MFS ↑Grade	[109]
	miR-23a		↓RFS	[66]
	miR-423		↑Metastasis	[132]
	miR-9		↑Grade, metastasis, LR	[87]
	miR-187		↓DSS, RFS	[133]
	miR-155		↑TNM, grade, LNM ↓OS	[134]
	miR-221/222		↓MFS	[135]
	miR-421, -486, -503, -720, -1303		↓MFS	[136]
	miR-375		↑LR	[137]
	miR-548 family		↑LNM ↓MFS	[95]
	miR-146a-5p		↓OS	[80]
	miR-27b-3p	TNBC tissues	↑Metastasis ↓DSS	[138]
	miR-93		↑LNM, TNM, grade, Ki-67	[139]
	miR-21, -210, -221		↓DFS, OS	[140]
	miR-34b			[141]
	miR-18b, -103, -107, -652	TNBC patients serum	↓OS, RFS.	[142]
	miR-200b	BC patients plasma	↓PFS, OS	[86]
	miR-202	BC patients serum	↓OS	[143]
	miR-10b-5p		↑TNM, grade, LNM	[144]
	miR-122		↓MFS, RFS	[78]
	miR-10b, -34a, -155		↑Metastasis	[145]

^↑^Incresead; ^↓^ Decreased

*RFS* relapse-free survival, *TNM* TNM classification of malignant tumours, *MFS* metastasis-free survival, *LR* local recurrence, *DSS* disease-specific survival

### Predictive biomarkers

The role of miRNAs as potential predictive biomarkers is also a field of growing interest. When investigating the regulation of miRNAs expression by antiestrogen therapies in human BC specimens using the initial biopsy and comparing it with the surgery specimen after neoadjuvant ET, Maillot and co-workers [146] noticed that some miRNAs that were previously shown overexpressed in tamoxifen-resistant cell lines were up-regulated following ET. These results highlight the utility of considering miRNA expression in understanding ET resistance in BC. Other studies have searched for miRNAs able to predict therapeutic response of BC patients to ET. For instance, Rodriguez-Gonzalez and colleagues [147] have found that miR-30c independently predicted clinical benefit of tamoxifen therapy in patients with advanced BC. On the other hand, Rothe et al. [110] found that miR-210 high level expressions were associated with a higher risk of recurrence in tamoxifen treated patients.

In addition to ET, miRNAs have been involved in responsiveness to other therapies. For instance, high circulating levels of miR-210 have been associated with resistance to anti-HER2 therapy using trastuzumab [148] and miR-100 expression has been positively correlated with sensitivity to chemotherapy using paclitaxel [119]. The potential role of miRNAs in the prediction of the response to these and other therapies, such as radiotherapy, are summarized in Table 4.

**Table 4:** miRNAs involved in therapeutic response (sensitivity/resistance) in BC

Therapies	Role in response	miRNA	Putative targets	Agent	Biological samples	Refs.
*Hormone therapy*						
Antiestrogens	Sensitivity	miR-342	CCNB1	Tamoxifen	Cell lines and tumor tissues	[149]
		miR-26a	EZH2		Tumor tissues	[150]
		miR-30c	EGFR			[147]
		miR-10, -126	–			[151]
	Resistance	miR-221/222	CDKN1B	Tamoxifen, fulvestrant	Tumor tissues and cell lines	[152, 153]
		miR-519a	CDKN1B PTEN, RB1	Tamoxifen	Tumor tissues and cell lines	[154]
		miR-155	SOCS6			[131]
		miR-210	–		Tumor tissues	[110]
		miR-301	FOXF2, BBC3, PTEN, COL2A1		Tumor tissues, cell lines and xenografts	[94]
Aromatase inhibitors	Sensitivity	miR-125b let-7c	ERBB2	Letrozole, anastrozole	Tumor tissues and cell lines	[116]
	Resistance	miR-181a	BCL2L11	Letrozole	Cell lines, xenografts and tumor tissues	[155]
Antibodies	Sensitivity	miR-210	–	Trastuzumab	Cell lines and plasma	[148]
	Resistance	miR-21	PTEN	Trastuzumab	Cell lines, xenograft and tumor tissues	[156]
Chemotherapy	Sensitivity	miR-451	MRP-1	Doxorubicin	Cell lines and tumor tissues	[157]
		miR-200c	MRP-1			[158]
		miR-134	ABCC1			[159]
		miR-128	BMI1, ABCC5	Doxorubicin, Paclitaxel	Cell lines, xenografts and tumor tissues	[121]
		miR-100	MTOR	Paclitaxel	Cell lines, xenografts and luminal tumor tissues	[119]
		miR-16	IKBKB		Cell lines and tumor tissues	[160]
		miR-621	FBXO11	Paclitaxel + Carboplatin	Cell lines, xenografts and tumor tissues	[161]
	Resistance	miR-125b	BAK1, E2F3	FEC	Cell lines, tumor tissues and serum	[162]
		miR-141	−	Taxane, Anthracyclines	Cell lines and tumor tissues	[163]
		miR-221	CDKN1B		Plasma	[164]
		miR-155	FOXO3a	Paclitaxel, VP-16, Doxorubicin	Cell lines and tumor tissues	[165]
Radiotherapy	Sensitivity	miR-155	RAD51	–	Cell lines and TNBC tissues	[166]

*CCNB1* cyclin B1, *EZH2* enhancer of zeste homolog 2, *EGFR* epidermal growth factor receptor, *CDKN1B* cyclin-dependent kinase inhibitor 1B, *PTEN* phosphatase and tensin homolog, *RB1* retinoblastoma 1, *SOCS6* suppressor of cytokine signaling 6, *FOXF2* forkhead box F2, *BBC3* BCL2 binding component 3, *COL2A1* collagen type II alpha 1, *ERBB2* Erb-B2 receptor tyrosine kinase 2, *BCL2L11* Bcl-2-like protein 11, *MRP1* multidrug resistance-associated protein 1, *ABCC1* ATP binding cassette subfamily C member 1, *BMI1* BMI1 polycomb ring finger oncogene, *ABCC5* ATP binding cassette subfamily C member 5, *MTOR* mechanistic target of rapamycin, *IKBKB* IκB kinase β, *FBXO11* F-box protein 11, *BAK1* BCL2 antagonist/killer 1, *E3F3* E2F transcription factor 3, *FOXO3a* Forkhead box O3a, *RAD51* RAD51 recombinase, FEC 5-florouracil, epirubucin and cyclophosphamide

Several clinical trials, summarized in Table 5, are currently ongoing to address the role of miRNAs in diagnosis, prognosis and prediction of response to therapy, aiming at the translation of current knowledge on miRNAs in BC into clinical practice.

**Table 5:** Ongoing clinical trials aiming at the introduction of miRNAs in clinical practice

Clinical trial	Patient population	Intervention	Aims	Study start date
NCT00581750 observational	Patients with lobular carcinoma in situ	Tumor profiling	Diagnosis	October 2001
NCT01231386 observational	Patients undergoing neoadjuvant or adjuvant chemotherapy and HT for locally advanced and inflammatory BC	Tumor profiling, Circulating miRNAs	Prognosis drug sensitivity	October 2014
NCT01722851 observational	Newly diagnosed BC patients who are scheduled to undergo neoadjuvant chemotherapy/HT and patients who present with disease recurrence or disease progression, and who are commenced on systemic therapies (HT and/or chemotherapy)	Circulating miRNAs	Prognosis drug sensitivity	September 2013
NCT02656589 observational	Patients with HER2^+^ advanced stage BC who were treated with herceptin		Drug sensitivity	June 2015
NCT01598285 observational	Patients suffering from metastatic BC, treated with bevacizumab			May 2012
NCT01612871 observational	Patients with metastatic invasive BC or locally advanced BC for which treatment with tamoxifen or anti-aromatase is indicated			June 2012

## Conclusion

BC is a very heterogeneous disease, and several biological features are routinely used for diagnostic, prognostic and predictive purposes, including histological grade, lymph node status, hormone receptor status, and HER2 status. These factors have been associated with BC patient’s survival and clinical outcome following treatment. Nevertheless, some patients with similar combination of those features follow different clinical paths, demonstrating that the capacity of determining prognosis and predicting therapeutic outcome in BC patients remains limited. Several mRNA-based tests are currently available with the aim of improving BC prognostication, but its use in clinical practice is still limited. New biomarkers are therefore needed to assist in improving BC patient prognostication and monitoring, allowing for a more precise definition of the therapeutic and follow-up strategy in an individual basis.

Based on the studies cited in this review, it is remarkable that ncRNAs are adding an extra dimension to the understanding of BC biology. miRNAs, in particular, are emerging as promising biomarkers for BC diagnosis (e.g. miR-155 and miR-195), prognosis (e.g. miR-29b and miR-30 family) and prediction of response to therapy (e.g. miR-30c and miR-221). It should be emphasized that miRNAs are easily accessible, affordable, non-invasive tools for personalized management of BC patients, since they circulate stably in bodily fluids. These features allow miRNAs to respond to current clinical needs and provide the opportunity to bypass the problems associated with tumor tissue biopsy. Although some lncRNAs have also shown potential to serve as BC biomarkers, the stability and origin of circulating lncRNAs remain largely unknown, and additional studies are required to support a definitive clinical application. Regarding tRNAs, many questions also remain unanswered, such as the origin and its physiological role.

When reviewing the data from several studies, widespread inconsistencies across them are found. The cause might be attributable to differences in sample type, with some studies using plasma or serum and other using whole blood, differences in technology platforms used for miRNA profiling, such as next-generation sequencing (NGS) or real time reverse transcription polymerase chain reaction, differences in the choice of pre- or—post-operative samples, as well as from the choice of different genes for data normalization. These discrepancies among reported signatures highlight the need to standardize experimental conditions for circulating miRNAs studies, as well as the need to validate these findings in additional independent cohorts as well as preclinical/clinical verification studies, before the clinical utility of circulating miRNAs may be established.

In conclusion, the emergence of ncRNA classes as possible BC biomarkers, mainly miRNAs, shows great potential to foster precision medicine in BC, although its application in clinical routine is still a long term goal.

## Abbreviations

BC: breast cancer
BCAR4: BC anti-estrogen resistance 4
CCAT2: lncRNA colon cancer associated transcript 2
CMF: cyclophosphamide, methotrexate and fluorouracil
DFS: disease-free survival
EGOT: eosinophil granule ontogeny transcript
ER: estrogen receptor
PR: progesterone receptor
HER2: human epidermal growth factor receptor 2
ET: endrocrine therapy
HOTAIR: HOX transcript antisense RNA
HT: hormone-therapies
LincRNAs: long intergenic non-coding RNAs
lncRNAs: long noncoding RNAs
LNM: lymph node metastasis
MALAT1: metastasis-associated lung adenocarcinoma transcript 1
miRNAs: microRNAs
mRNA: messenger RNA
NBAT1: neuroblastoma associated transcript 1
ncRNAs: noncoding RNAs
OncomiRs: oncogenic miRNAs
OS: overall survival
PFS: progression free-survival
PTPRG-AS1: PTPRG antisense RNA 1
ROR: regulator of reprogramming
rRNAs: ribosomal RNAs
SPRY4-IT1: SPRY4 intronic transcript 1
SNHG17: small nucleolar RNA host gene 17
snoRNAs: small nucleolar RNAs
tRNAs: transfer RNAs
